# Effect of Humanin G (HNG) on inflammation in age-related macular degeneration (AMD)

**DOI:** 10.18632/aging.204074

**Published:** 2022-05-16

**Authors:** Sonali Nashine, Pinchas Cohen, Junxiang Wan, M. Cristina Kenney

**Affiliations:** 1 Department of Ophthalmology, Gavin Herbert Eye Institute, University of California Irvine, Irvine, CA 92697, USA; 2 Davis School of Gerontology, University of Southern California, Los Angeles, CA 90007, USA; 3 Department of Pathology and Laboratory Medicine, University of California Irvine, Irvine, CA 92697, USA

**Keywords:** Humanin G, HNG, AMD, inflammation, age-related macular degeneration

## Abstract

Inflammation plays a crucial role in the etiology and pathogenesis of AMD (Age-related Macular Degeneration). Humanin G (HNG) is a Mitochondrial Derived Peptide (MDP) that is cytoprotective in AMD and can protect against mitochondrial and cellular stress induced by damaged AMD mitochondria. The goal of this study was to test our hypothesis that inflammation-associated marker protein levels are increased in AMD and treatment with HNG leads to reduction in their protein levels. Humanin protein levels were measured in the plasma of AMD patients and normal subjects using ELISA assay. Humanin G was added to AMD and normal (control) cybrids which had identical nuclei from mitochondria-deficient ARPE-19 cells but differed in mitochondrial DNA (mtDNA) content derived from clinically characterized AMD patients and normal (control) subjects. Cell lysates were extracted from untreated and HNG-treated AMD and normal cybrids, and the Luminex XMAP multiplex assay was used to measure the levels of inflammatory proteins. AMD plasma showed reduced Humanin protein levels, but higher protein levels of inflammation markers compared to control plasma samples. In AMD RPE cybrid cells, Humanin G reduced the CD62E/ E-Selectin, CD62P/ P-Selectin, ICAM-1, TNF-α, MIP-1α, IFN–γ, IL-1β, IL-13, and IL-17A protein levels, thereby suggesting that Humanin G may rescue from mtDNA-mediated inflammation in AMD cybrids. In conclusion, we present novel findings that: A) show reduced Humanin protein levels in AMD plasma vs. normal plasma; B) suggest the role of inflammatory markers in AMD pathogenesis, and C) highlight the positive effects of Humanin G in reducing inflammation in AMD.

## INTRODUCTION

Although the immune system plays a pivotal role in maintaining cellular and tissue homeostasis and provides a critical defense mechanism against insults, immune dysregulation that causes sustained chronic low-grade inflammation leads to inflammatory disorders and is detrimental [1].

Aging is often accompanied by immunosenescence and immune dysregulation, which is characterized by aberrant apoptotic ability, enhanced basal inflammation, an imbalance in the proinflammatory and anti-inflammatory factors, and diminished ability to mount adequate immune responses to new pathogens. Aged adults have higher blood levels of inflammation markers than the normal baseline levels found in younger adults [2].

The eye is an immune-privileged organ and therefore immune responses to foreign antigens are suppressed or inhibited. The eye is protected by anatomical barriers such as the blood-retinal barrier formed by tight junctions of the endothelial cells and also the retinal pigment epithelial (RPE) cells. Furthermore, retinal neurons, RPE cells, and ocular fluids express immune modulators that can suppress activation of complement, microglial cells, and macrophages. However, acute insults contribute to retinal inflammation and subsequent retinal degeneration [3]. Inflammatory processes, primarily components of innate immunity including the complement system, microglia, macrophages, cytokines, and chemokines drive the progression of age-related macular degeneration (AMD) disease that is a leading cause of vision loss in the United States [4, 5]. A clinical hallmark of AMD is the formation of drusen deposits between the RPE cells and Bruch’s membrane, and drusen deposits are composed of inflammatory cytokines and complement proteins [6–8]. Cigarette smoking triggers the secretion and activation of proinflammatory cytokines and causes immune suppressive effects [9]. Recruitment of macrophages at the choroid leads to choroidal inflammation and abnormal blood vessel growth, thereby contributing to choroidal neovascularization in wet AMD. Macrophage polarization determines the potential of macrophages to regulate angiogenesis [10, 11]. AMD pathology is characterized by activation of inflammatory cytokines that in turn influences the polarization of macrophages. Furthermore, the infiltration of retinal microglia into the outer retina in the aged eye results in interaction of RPE cells with microglia, which in turn causes critical structural and physiological changes in the RPE, thereby contributing to neuroinflammation in AMD [12, 13].

Since inflammatory cells play a key role in the pathogenesis and progression of AMD, several therapeutic interventions for AMD have focused on reducing chronic inflammation to delay or prevent retinal degeneration in AMD [14, 15]. However, to our knowledge, no previous study has investigated the role of Humanin G, a S14G variant of Humanin, which is a cytoprotective mitochondrial-derived peptide that protects against RPE cell death in AMD [16, 17]. Therefore, in this study, we compared the protein levels of inflammation markers and investigated the effects of treatment with exogenous Humanin G in normal and AMD RPE transmitochondrial cybrid cells. We found differential levels of inflammation proteins between normal and AMD plasma samples and observed that treatment with Humanin G markedly reduced the protein levels of inflammation markers that were elevated in AMD RPE transmitochondrial cybrid cells. Our discovery is novel and may contribute to the development of therapeutics/ tools for reducing inflammation to alleviate AMD disease pathology.

## MATERIALS AND METHODS

### Human subjects

The University of California Irvine’s IRB (Institutional Review Board) approved research with human subjects (Approval #2003–3131). All participants provided informed consent and clinical investigations were performed according to the tenets of Declaration of Helsinki.

### Cell culture

Normal and AMD ARPE-19 transmitochondrial cybrid cell lines were created as described previously [8, 18–23]. Briefly, these cybrid cell lines were prepared by polyethylene glycol fusion of mitochondria DNA-deficient ARPE-19 (Rho0) cell line with platelets isolated from normal subjects and AMD patients. Cybrid status and that the cybrids have acquired their mtDNAs from the donor individuals were confirmed using allelic discrimination, Sanger sequencing, and Next-Generation Sequencing. The base medium for cybrid cell lines is DMEM-F12 Medium (Cat. # 10-092CM, Fisher Scientific, Pittsburgh, PA, USA). DMEM-F12 Medium contains 3.15 g/L D-glucose, 2.5 mM L-glutamine, 15 mM HEPES, 0.5 mM sodium pyruvate, and 1200 mg/L sodium bicarbonate. To make the complete growth medium, fetal bovine serum was added to the base medium to a final concentration of 10%.

### ELISA measurement

Humanin levels of plasma were measured by in-house humanin ELISA developed at UCLA. The rabbit anti-human analogue HNG polyclonal anti-sera were produced at Harlan Laboratories (Indianapolis, IN, USA). IgG subclasses purified with a protein A column chromatography (Pierce Chemicals, Rockford, IL, USA) were used as capture antibodies. IgG was further purified with a Humanin G-conjugated ligand affinity column and labeled with biotin. This biotinylated ligand affinity purified anti-Humanin G IgG was used as detection antibody. To measure endogenous Humanin levels, synthetic Humanin purchased from Bachem (Torrance, CA, USA) was used as standard within the range of 0.1 ng/ml to 50 ng/ml. The intra- and inter-assay coefficient variations (CV) were less than 10%. Prior to assay, humanin was extracted in 90% acetonitrile and 10% 1N HCl. Briefly, 200 μl of extraction reagent was added to 100 μl of plasma or protein extracts, gently mixed and incubated at room temperature for 30 min. The mixture was centrifuged, and the supernatant was removed and dried. The dried extract was reconstituted with 200 μl of phosphate buffer (50 mM sodium phosphate, 150 mM sodium chloride, 0.5% Tween-20, pH 7.6) and then used for assay. Ninety-six-well microtiter plates were coated with capture antibody at 0.5 μg/well in 200 μl of 50 mM sodium bicarbonate buffer, pH 9.5, incubated 3-4 h at room temperature on a shaker, and then washed with wash buffer followed by 2 washes with Superblock buffer (Pierce Chemicals, Rockford, IL, USA). Standards, controls or extracted samples, and pre-titered detection antibody were added to the appropriate wells and incubated overnight. Wells were then washed followed by addition of streptavidin-HRP conjugate and further incubation for 30 min at room temperature. After 4 washes with wash buffer, 200 μl/well of o-phenylenediamine hydrochloride solution (1 mg/mL in hydrogen peroxide substrate) was added and incubated for 10-20 min. The reaction was stopped by the addition of 50 μl/well 2N H_2_SO_4_ and absorbance was measured on a plate spectrophotometer (Molecular Designs, Sunnyvale, CA, USA) at 490 nm.

### Treatment with Humanin G (HNG)

Lyophilized HNG (Anaspec, Fremont, CA, USA) was reconstituted in water to obtain a stock solution that was subsequently dissolved in culture media to obtain a 3.2 μM HNG working solution. In this study, all cybrids were treated with 3.2 μM HNG. At the beginning of our initial study with HNG, we tested a wide range of concentrations (based on the published Humanin literature) ranging from 0.8 to 10 μM to determine a low optimum working concentration of HNG in our ARPE-19 cybrid cells. The HNG concentrations of 0.8 and 1.6 μM did not show any protective effects. Our preliminary studies demonstrated that 3.2 μM was the lowest concentration that showed significant protective effects in the cybrid cells. Therefore, we used 3.2 μM as our final working concentration for all experiments with HNG. HNG treatment timepoint was 48 h.

### Protein extraction

Normal and AMD cybrid cells were plated in six well plates, treated with Humanin G followed by incubation at 37° C. Cells were then lysed using RIPA buffer (Life Technologies, Carlsbad, CA, USA), supernatants were transferred to a new microfuge tube, and concentrations of proteins were measured using Bio-Rad Dc protein assay system (Bio-Rad Laboratories, Richmond, CA, USA) according to the manufacturer's instructions.

### Luminex protein assay

Luminex assay is essentially a bead-based sandwich immunoassay that allows simultaneous detection of multiple analytes in a sample. Using the Luminex xMAP technology and MAGPIX (Luminex Corporation, Austin, TX, USA) instrument, we tested markers of inflammation pathways in the normal and AMD ARPE-19 transmitochondrial cybrid cell lines. Inflammation Human Panel was purchased from Thermo Fisher Scientific (Waltham, MA, USA) and experiments were performed as per the manufacturer’s protocol. Briefly, the Luminex beads are internally dyed with precise proportions of red and infrared fluorophores to create spectrally unique signatures that can be identified by the Luminex xMAP detection systems (e.g., MAGPIX). This assay uses matched antibody pairs to identify the analyte i.e., the protein of interest. In a multiplexed assay, each spectrally unique bead is labeled with antibodies specific for a single target protein, and bound proteins are identified with biotinylated antibodies and streptavidin–R-phycoerythrin. The conjugation of protein-specific antibodies to a distinct bead allows for analysis of multiple targets in a single well. For detection, the MAGPIX xMAP instrument contains two lasers, one to distinguish the spectral signature of each bead and the second to determine the magnitude of streptavidin–R-phycoerythrin fluorescence, which is proportional to the amount of analyte bound. Multiplexing reduces costs and delivers faster, more reproducible results.

### Statistical analysis

Results between groups were analyzed for differences using the unpaired non-parametric Mann-Whitney test (GraphPad Prism 5.0; GraphPad Software, CA, USA). Statistical significance was determined at P-values < 0.05.

## RESULTS

### Humanin protein levels in normal and AMD plasma

**Figure 1:**
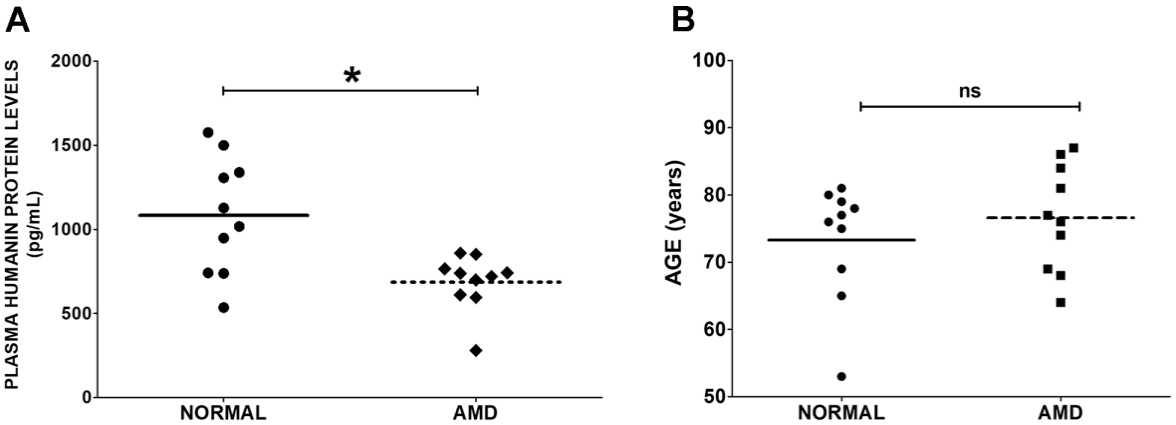
**Humanin levels in plasma.** (**A**) Humanin protein level was measured using ELISA assay. AMD patients had significantly reduced levels of Humanin protein compared to normal (control) subjects. (**B**) The difference in mean ages between normal subjects vs. AMD patients was non-significant. Data are presented as mean ± SEM. * P<0.05; ns: non-significant.

To determine the levels of Humanin protein in the plasma from AMD patients and normal subjects, ELISA assay was performed. We found that Humanin protein levels were reduced by 36.58 % (P = 0.0155) in the plasma from AMD patients: 686.1 ± 52.67 (Mean ± SEM) pg/mL; n=10, compared to that in the plasma from age-matched normal subjects: 1082 ± 110.1 pg/mL; n=10 (Figure 1A). We found no significant difference in the mean ages of normal subjects vs. AMD patients: Normal: 73.30 ± 2.753 years; AMD: 76.60 ± 2.513 years; P = 0.5702 (Figure 1B).

### Differential expression of inflammation markers between normal and AMD and effects of exogenous Humanin G on inflammation proteins

To compare the protein levels of inflammation markers between: A) normal plasma (n=3-8) and AMD plasma (n=3-8), and B) untreated vs. HNG-treated normal (n=4-7) and AMD (n=4-7) RPE cybrid cells, we used the Luminex xMAP technology.

**Figure 2:**
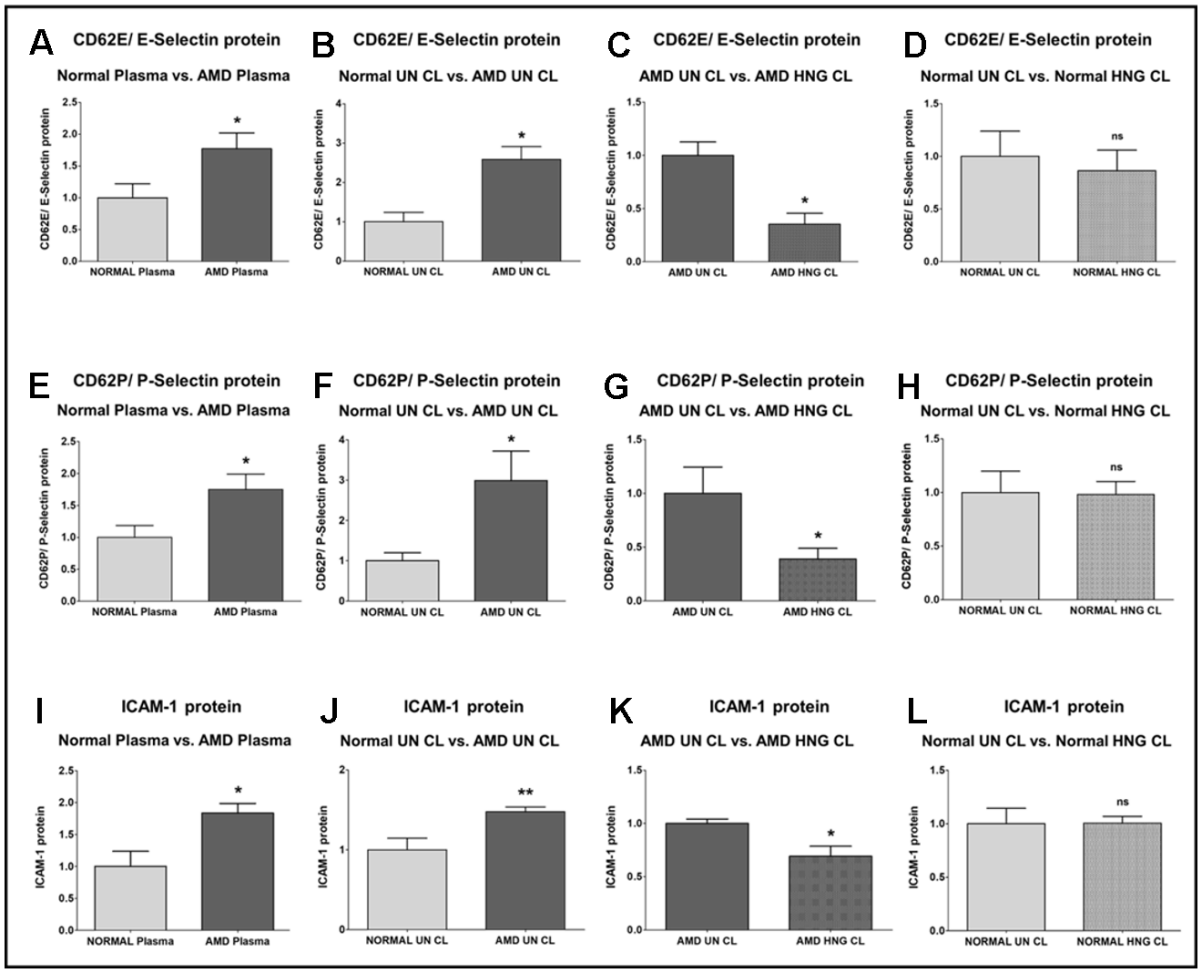
**Effect of Humanin G (HNG) on CD62E/E-Selectin, CD62P/P-Selectin, and ICAM-1 proteins.** CD62E/E-Selectin protein was increased significantly in AMD plasma (**A**) and in AMD RPE cybrid cells (**B**) compared to their normal counterparts. Treatment with HNG reduced CD62E/E-Selectin protein levels in AMD RPE cybrid ells (**C**) but not in normal RPE cybrid cells (**D**), compared to their untreated counterparts. CD62P/P-Selectin protein was elevated in AMD plasma (**E**) and in AMD RPE cybrid cells (**F**) compared to their normal counterparts. Treatment with HNG reduced CD62P/P-Selectin protein levels in AMD RPE cybrid ells (**G**) but not in normal RPE cybrid cells (**H**), compared to their untreated counterparts. ICAM-1 protein was elevated in AMD plasma (**I**) and in AMD RPE cybrid cells (**J**) compared to their normal counterparts. Treatment with HNG reduced ICAM-1 protein levels in AMD RPE cybrid ells (**K**) but not in normal RPE cybrid cells (**L**), compared to their untreated counterparts. Data are presented as mean ± SEM. * P<0.05; ** P<0.01; ns: non-significant.

We found that E-Selectin *(Endothelial-Selectin)*/ CD62E (*CD62 (Cluster of Differentiation 62) antigen-like family member E*) protein level was significantly higher by 77.1% in AMD plasma compared to normal plasma: P = 0.0293; Normal plasma = 1 ± 0.2215 a.u. (arbitrary units); AMD plasma = 1.771 ± 0.2484 a.u. (Figure 2A). Untreated AMD cell lysates also showed 158.5 % higher CD62E/E-Selectin protein levels compared to untreated normal cell lysates: P = 0.0129; Normal untreated cell lysate (NL UN CL) = 1 ± 0.2392 a.u.; AMD untreated cell lysate (AMD UN CL) = 2.585 ± 0.3239 a.u. (Figure 2B). Treatment with HNG reduced CD62E/E-Selectin protein levels by 64.62 % in AMD cells: P = 0.0129; AMD UN CL = 1 ± 0.1253 a.u.; AMD HNG-treated cell lysate (AMD HNG CL) = 0.3538 ± 0.1020 a.u. (Figure 2C). Addition of HNG to normal cells reduced CD62E/E-Selectin protein levels by 13.8 %, but the difference was non-significant: P = 0.7479; Normal UN CL = 1 ± 0.2392 a.u.; NL HNG CL = 0.8620 ± 0.1972 a.u. (Figure 2D).

P-Selectin *(Platelet-Selectin)*/ CD62P (*CD62 antigen-like family member P*) protein level was significantly higher by 75 % in AMD plasma compared to normal plasma: P = 0.0426; Normal plasma = 1 ± 0.1853 a.u.; AMD plasma = 1.750 ± 0.2407 a.u. (Figure 2E). Untreated AMD cell lysates also showed 198.9 % higher CD62P/P-Selectin protein levels compared to untreated normal cell lysates: P = 0.0317; NL UN CL = 1 ± 0.1980 a.u.; AMD UN CL = 2.989 ± 0.7296 a.u. (Figure 2F). Treatment with HNG reduced CD62P/P-Selectin protein levels by 60.99 % in AMD cells: P = 0.0420; AMD UN CL = 1 ± 0.2441 a.u.; AMD HNG CL = 0.3901 ± 0.09880 a.u. (Figure 2G). Addition of HNG to normal cells reduced CD62P/ P-Selectin protein levels by 1.94 %, but the difference was non-significant: P = 0.9347; Normal UN CL = 1 ± 0.1980 a.u.; NL HNG CL = 0.9806 ± 0.1225 a.u. (Figure 2H).

ICAM-1 (*Intercellular Adhesion Molecule-1*) protein was significantly increased by 83.6 % in AMD plasma compared to normal plasma: P = 0.0177; Normal plasma = 1 ± 0.2365 a.u.; AMD plasma = 1.836 ± 0.1512 a.u. (Figure 2I). Untreated AMD cell lysates also showed 47.5 % higher ICAM-1 protein levels compared to untreated normal cell lysates: P = 0.0079; NL UN CL = 1 ± 0.1453 a.u.; AMD UN CL = 1.475 ± 0.06149 a.u. (Figure 2J). Treatment with HNG reduced ICAM-1 protein levels by 30.82 % in AMD cells: P = 0.0303; AMD UN CL = 1 ± 0.04168 a.u.; AMD HNG CL = 0.6918 ± 0.09460 a.u. (Figure 2K). Addition of HNG to normal cells showed no change in ICAM-1 protein levels: P = 0.5368; Normal UN CL = 1 ± 0.1453 a.u.; NL HNG CL = 1 ± 0.06405 a.u. (Figure 2L).

**Figure 3:**
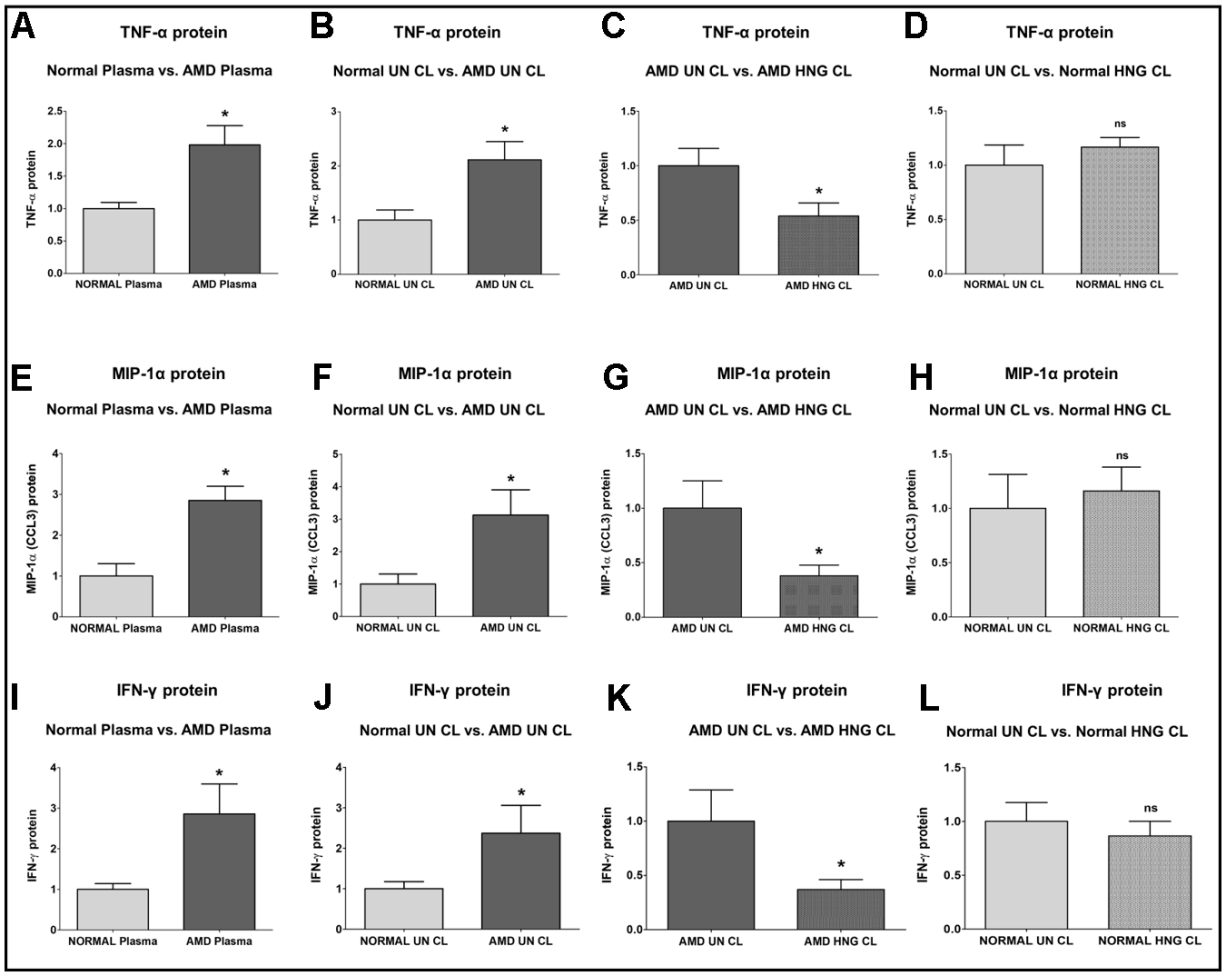
**Effect of Humanin G (HNG) on TNF-α, MIP-1α, and IFN-γ proteins.** TNF-α protein was elevated in AMD plasma (**A**) and in AMD RPE cybrid cells (**B**) compared to their normal counterparts. Treatment with HNG reduced TNF-α protein levels in AMD RPE cybrid ells (**C**) but not in normal RPE cybrid cells (**D**), compared to their untreated counterparts. MIP-1α protein was elevated in AMD plasma (**E**) and in AMD RPE cybrid cells (**F**) compared to their normal counterparts. Treatment with HNG reduced MIP-1α protein levels in AMD RPE cybrid ells (**G**) but not in normal RPE cybrid cells (**H**), compared to their untreated counterparts. IFN-γ protein was elevated in AMD plasma (**I**) and in AMD RPE cybrid cells (**J**) compared to their normal counterparts. Treatment with HNG reduced IFN-γ protein levels in AMD RPE cybrid cells (**K**) but not in normal RPE cybrid cells (**L**), compared to their untreated counterparts. Data are presented as mean ± SEM. * P<0.05; ns: non-significant.

TNF-α (*Tumor Necrosis Factor-α*) protein was significantly increased by 98.4 % in AMD plasma compared to normal plasma: P=0.0421; Normal plasma = 1 ± 0.09602 a.u.; AMD plasma = 1.984 ± 0.2939 a.u. (Figure 3A). Untreated AMD cell lysates also showed 111.3 % higher TNF-α protein levels compared to untreated normal cell lysates: P = 0.0286; NL UN CL = 1 ± 0.1870 a.u.; AMD UN CL = 2.113 ± 0.3367 a.u. (Figure 3B). Treatment with HNG reduced TNF-α protein levels by 46.09 % in AMD cells: P = 0.0381; AMD UN CL = 1 ± 0.1594 a.u.; AMD HNG CL = 0.5391 ± 0.1198 a.u. (Figure 3C). Addition of HNG to normal cells caused no difference in TNF-α protein levels: P = 0.8857; Normal UN CL = 1 ± 0.1870 a.u.; NL HNG CL = 1.045 ± 0.05271 a.u. (Figure 3D).

MIP-1α (*Macrophage Inflammatory protein-1α*)/ CCL3 protein was increased by 185.2 % in AMD plasma compared to normal plasma: P = 0.0357; Normal plasma = 1 ± 0.2994 a.u.; AMD plasma = 2.852 ± 0.3444 a.u. (Figure 3E). Untreated AMD cell lysates also showed 212.2 % higher MIP-1α protein levels compared to untreated normal cell lysates: P = 0.0317; NL UN CL = 1 ± 0.3142 a.u.; AMD UN CL = 3.122 ± 0.7811 a.u. (Figure 3F). Treatment with HNG reduced MIP-1α protein levels by 61.98 % in AMD cells: P = 0.0173; AMD UN CL = 1 ± 0.2502 a.u.; AMD HNG CL = 0.3802 ± 0.09824 a.u. (Figure 3G). Addition of HNG to normal cells caused no change in MIP-1α protein levels: P = 0.6905; Normal UN CL = 1 ± 0.3142 a.u.; NL HNG CL = 1.014 ± 0.1991 a.u. (Figure 3H).

IFN-γ (*Interferon-gamma*) protein level was significantly higher by 186.1 % in AMD plasma compared to normal plasma: P = 0.0286; Normal plasma = 1 ± 0.1416 a.u.; AMD plasma = 2.861 ± 0.7355 a.u. (Figure 3I). Untreated AMD cell lysates also showed 137.3 % higher IFN-γ protein levels compared to untreated normal cell lysates: P = 0.0381; NL UN CL = 1 ± 0.1748 a.u.; AMD UN CL = 2.373 ± 0.6844 a.u. (Figure 3J). Treatment with HNG reduced IFN-γ protein levels by 62.86 % in AMD cells: P = 0.0381; AMD UN CL = 1 ± 0.2884 a.u.; AMD HNG CL = 0.3714 ± 0.09164 a.u. (Figure 3K). Addition of HNG to normal cells reduced IFN-γ protein levels by 13.5 %, but the difference was non-significant: P = 1; Normal UN CL = 1 ± 0.1748 a.u.; NL HNG CL = 0.8650 ± 0.1353 a.u. (Figure 3L).

**Figure 4:**
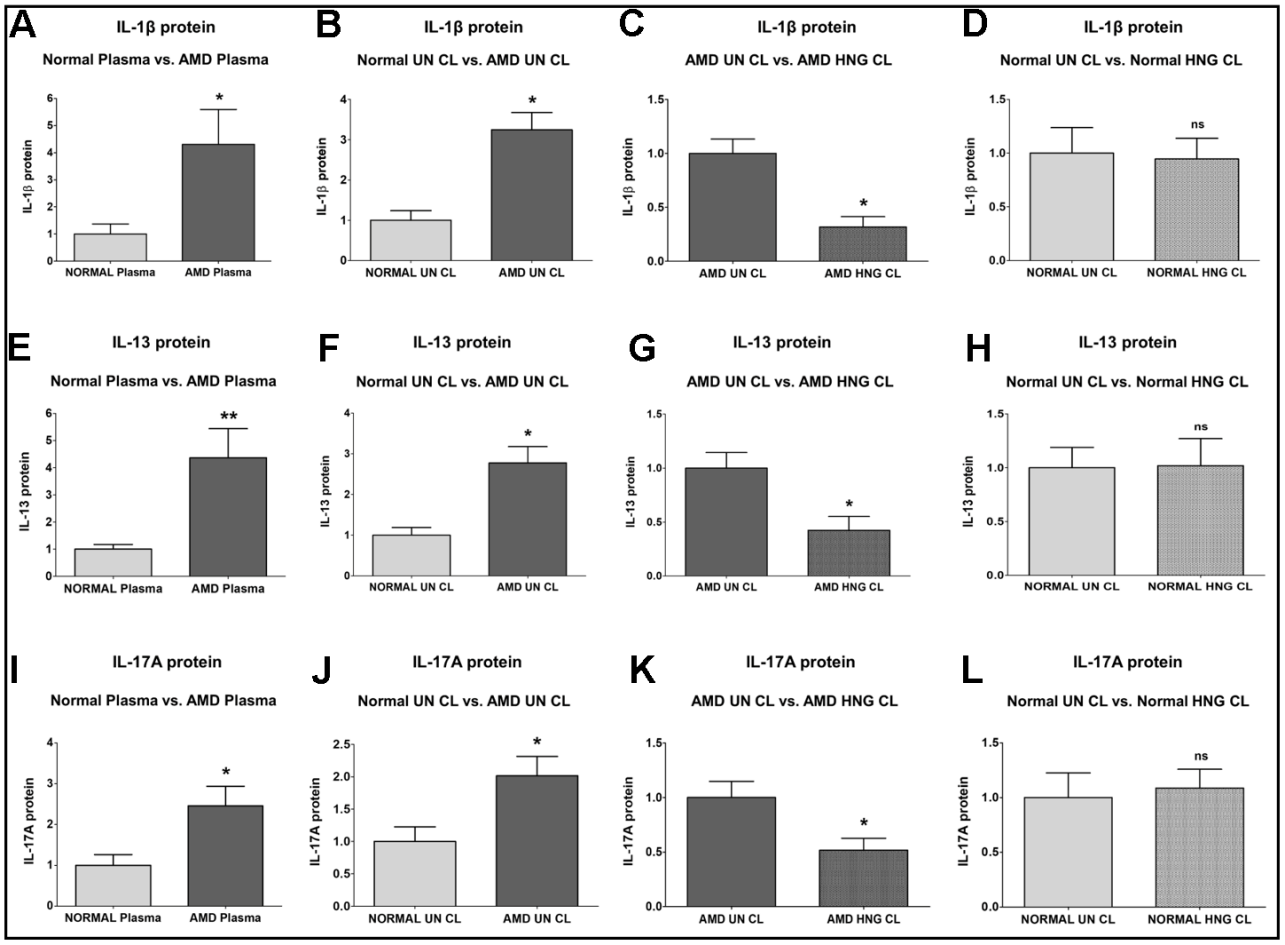
**Effect of Humanin G (HNG) on IL-1β, IL-13, and IL-17A proteins.** IL-1β protein was elevated in AMD plasma (**A**) and in AMD RPE cybrid cells (**B**) compared to their normal counterparts. Treatment with HNG reduced IL-1β protein levels in AMD RPE cybrid ells (**C**) but not in normal RPE cybrid cells (**D**), compared to their untreated counterparts. IL-13 protein was elevated in AMD plasma (**E**) and in AMD RPE cybrid cells (**F**) compared to their normal counterparts. Treatment with HNG reduced IL-13 protein levels in AMD RPE cybrid ells (**G**) but not in normal RPE cybrid cells (**H**), compared to their untreated counterparts. IL-17A protein was elevated in AMD plasma (**I**) and in AMD RPE cybrid cells (**J**) compared to their normal counterparts. Treatment with HNG reduced IL-17A protein levels in AMD RPE cybrid ells (**K**) but not in normal RPE cybrid cells (**L**), compared to their untreated counterparts. Data are presented as mean ± SEM. * P<0.05; ** P<0.01; ns: non-significant.

IL-1β (*Interleukin-1β*) protein was significantly increased by 330.6 % in AMD plasma compared to normal plasma: P = 0.0286; Normal plasma = 1 ± 0.3704 a.u.; AMD plasma = 4.306 ± 1.289 a.u. (Figure 4A). Untreated AMD cell lysates also showed 224.5 % higher IL-1β protein levels compared to untreated normal cell lysates: P = 0.0286; NL UN CL = 1 ± 0.2377 a.u.; AMD UN CL = 3.245 ± 0.4314 a.u. (Figure 4B). Treatment with HNG reduced IL-1β protein levels by 68.31 % in AMD cells: P = 0.0286; AMD UN CL = 1 ± 0.1330 a.u.; AMD HNG CL = 0.3169 ± 0.09678 a.u. (Figure 4C). Addition of HNG to normal cells reduced IL-1β protein levels by 5.45 %, but the difference was non-significant: P = 0.8857; Normal UN CL = 1 ± 0.2377 a.u.; NL HNG CL = 0.9455 ± 0.1937 a.u. (Figure 4D).

IL-13 (*Interleukin-13*) protein was significantly increased by 336.5 % in AMD plasma compared to normal plasma: P = 0.0043; Normal plasma = 1 ± 0.1707 a.u.; AMD plasma = 4.365 ± 1.076 a.u. (Figure 4E). Untreated AMD cell lysates also showed 177.6 % higher IL-13 protein levels compared to untreated normal cell lysates: P = 0.0286; NL UN CL = 1 ± 0.1896 a.u.; AMD UN CL = 2.776 ± 0.4021 a.u. (Figure 4F). Treatment with HNG reduced IL-13 protein levels by 57.63 % in AMD cells: P = 0.0421; AMD UN CL = 1 ± 0.1448 a.u.; AMD HNG CL = 0.4237 ± 0.1280 a.u. (Figure 4G). Addition of HNG to normal cells did not cause any change in the IL-13 protein levels: P = 1; Normal UN CL = 1 ± 0.1896 a.u.; NL HNG CL = 1.020 ± 0.2515 a.u. (Figure 4H).

IL-17A (*Interleukin-17*) protein was significantly increased by 145.7 % in AMD plasma compared to normal plasma: P = 0.0317; Normal plasma = 1 ± 0.2619 a.u.; AMD plasma = 2.457 ± 0.4793 a.u. (Figure 4I). Untreated AMD cell lysates also showed 101.4 % higher IL-17A protein levels compared to untreated normal cell lysates: P = 0.0159; NL UN CL = 1 ± 0.2259 a.u.; AMD UN CL = 2.014 ± 0.2994 a.u. (Figure 4J). Treatment with HNG reduced IL-17A protein levels by 48.31 % in AMD cells: P = 0.0303; AMD UN CL = 1 ± 0.1487 a.u.; AMD HNG CL = 0.5169 ± 0.1098 a.u. (Figure 4K). Addition of HNG to normal cells caused no change in the IL-17A protein levels: P = 0.8413; Normal UN CL = 1 ± 0.2259 a.u.; NL HNG CL = 1.087 ± 0.1721 a.u. (Figure 4L).

**Figure 5:**
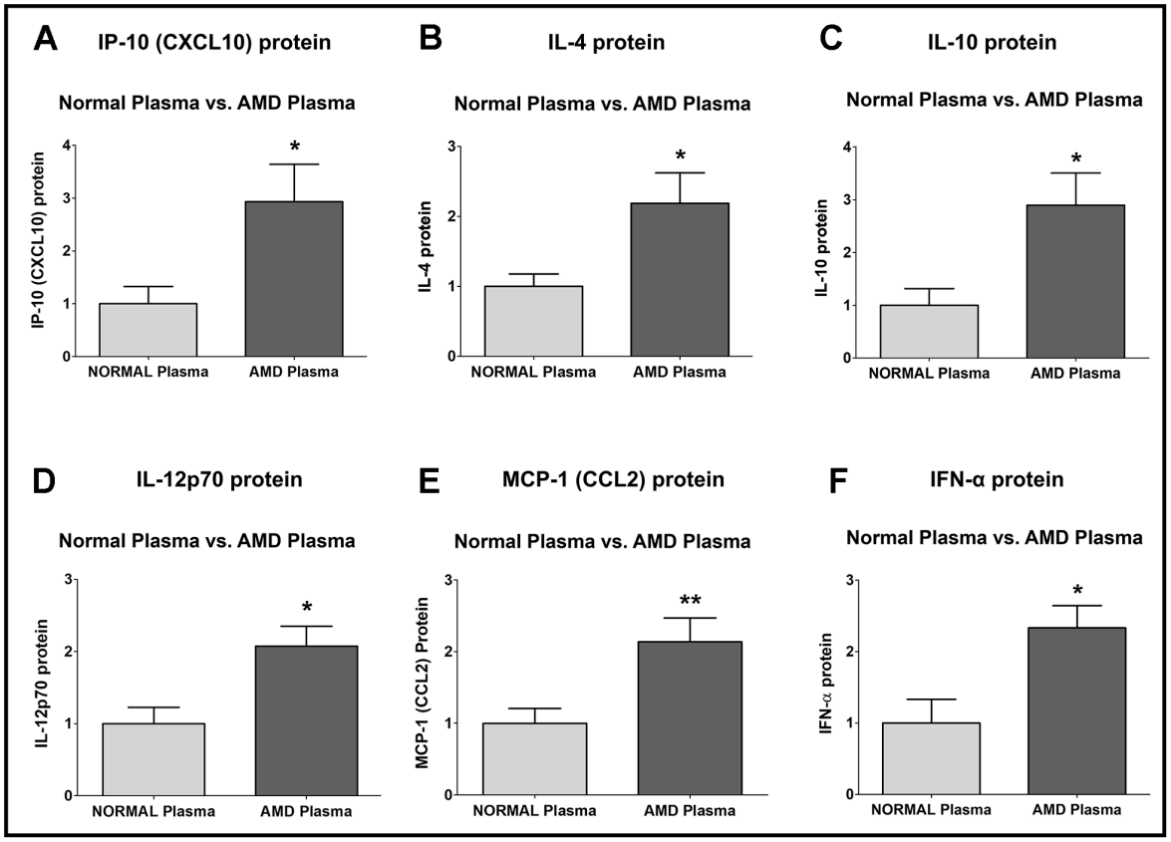
**Protein levels of IP-10, IL-4, IL-10, IL-12p70, MCP-1, and IFN-α.** IP-10 (**A**), IL-4 (**B**), IL-10 (**C**), IL-12p70 (**D**), MCP-1 (**E**), and IFN-α (**F**) protein levels were remarkably higher in AMD plasma compared to normal plasma. Data are presented as mean ± SEM. * P<0.05; ** P<0.01; ns: non-significant.

IP-10 (*Interferon-gamma-induced Protein 10*)/ CXCL10 (*C-X-C motif chemokine Ligand 10*) protein was significantly higher by 193.5 % in AMD plasma compared to normal plasma: P = 0.0286; Normal plasma = 1 ± 0.3277 a.u.; AMD plasma = 2.935 ± 0.7114 a.u. (Figure 5A).

IL-4 (*Interleukin-4*) protein was significantly higher by 118.5 % in AMD plasma compared to normal plasma: P = 0.0317; Normal plasma = 1 ± 0.1770 a.u.; AMD plasma = 2.185 ± 0.4348 a.u. (Figure 5B).

IL-10 (*Interleukin-10*) protein was significantly higher by 189.7 % in AMD plasma compared to normal plasma: P = 0.0159; Normal plasma = 1 ± 0.3180 a.u.; AMD plasma = 2.897 ± 0.6058 a.u. (Figure 5C).

IL-12p70 (*Interleukin-12, p70*) protein was significantly higher by 107.7 % in AMD plasma compared to normal plasma: P = 0.0101; Normal plasma = 1 ± 0.2275 a.u.; AMD plasma = 2.077 ± 0.2757 a.u. (Figure 5D).

MCP-1 (*Monocyte Chemoattractant Protein-1*)/ CCL2 (*Chemokine C-C motif Ligand 2*) protein was significantly higher by 113.5 % in AMD plasma compared to normal plasma: P = 0.0081; Normal plasma = 1 ± 0.2085 a.u.; AMD plasma = 2.135 ± 0.3358 a.u. (Figure 5E).

IFN-α (*Interferon-alpha*) protein was significantly higher by 133.3 % in AMD plasma compared to normal plasma: P = 0.0173; Normal plasma = 1 ± 0.3335 a.u.; AMD plasma = 2.333 ± 0.3108 a.u. (Figure 5F).

## DISCUSSION

In this study, we examined the levels of Humanin and inflammation proteins in plasma from AMD vs. normal groups and investigated the effects of Humanin G on inflammatory proteins in normal and AMD RPE transmitochondrial cybrid cells. We demonstrated significantly reduced Humanin protein levels in the plasma of AMD patients compared to that in the plasma of age-matched normal subjects that served as controls. In addition, we reported activation of inflammation markers in AMD plasma and AMD RPE cybrid cells compared to their normal/control counterparts. Furthermore, we demonstrated that treatment with exogenous Humanin G can revert the abnormal levels of inflammation proteins found in AMD samples to close to normal ranges. These findings are novel and significant as ocular inflammation contributes to retinal degeneration and is a crucial player in the etiology and pathogenesis of AMD [24].

The strong potential of the most conserved mitochondrial-derived peptide, Humanin and its more potent variant Humanin G in protecting damaged mitochondria and RPE cells is well-established in retinal diseases including AMD [17, 25, 26]. Our previous study with Humanin G showed that mitochondria from AMD patients are severely damaged and highlighted the protective role of Humanin G against mitochondria-mediated and amyloid-β-induced cell death in AMD transmitochondrial ARPE-19 cybrids. One mechanism by which Humanin G protected AMD cybrid cells from death was through stabilization of mitochondria and prevention of mitochondrial death. In that study, real-time PCR with primers spanning 503–2484 bps mtDNA regions showed increased numbers of mtDNA lesions in this region of the AMD mitochondrial genome [16].

In the current study, we found that the plasma levels of endogenous Humanin protein were significantly lower by 36.58 % in AMD patients compared to that in age-matched normal subjects. To our knowledge, this is the first study that has reported notably reduced Humanin protein levels in AMD patients, thereby corroborating the pivotal role of Humanin in maintaining tissue homeostasis and normal functioning in the eye. Our study is consistent with previous studies showing that aging is accompanied by markedly reduced Humanin levels and adequate Humanin levels are proportional to increased lifespan and better health, since reduced Humanin levels are observed in aging-related illnesses such as Alzheimer’s disease and diabetes [27, 28]. Along similar lines, Humanin protein levels are remarkably higher in the plasma and skeletal muscle of humans following high-intensity exercise and resistance training, indicating a role of Humanin in regulating glucose metabolism as well [29, 30]. With age, the amount of baseline Humanin decreases in the hypothalamus, cortex, and skeletal muscle [31]. Moreover, treatment with exogenous Humanin G is known to reduce the expression of markers associated with aging-related disorders [32]. Therefore, we tested the effects of Humanin G on inflammatory markers in this study.

During the course of inflammation, endothelial cell activation leads to the expression of cell adhesion molecules, which mediate the trafficking of inflammatory and immune cells toward the sites of inflammation and promote their interaction with the activated endothelium [33]. Selectins/CD62 are cell adhesion molecules that are cell surface C-type lectins and soluble transmembrane glycoproteins involved in acute and chronic inflammation processes and facilitate the adhesive process in inflammation by enabling leukocyte rolling on vascular surfaces [34–37]. E-Selectin i.e., CD62E/ ELAM-1 (Endothelial-Leukocyte Adhesion Molecule 1), is a 115 kDa endothelial cell surface specific adhesion molecule that is expressed by the *SELE* (Selectin E) gene in humans. It is composed of an N-terminal C-type lectin domain, an EGF (Epidermal-Growth-Factor)-like domain, a conserved protein domain of six short consensus repeats of cysteine residues, a transmembrane domain, and a cytoplasmic tail. The expression of E-Selectin on endothelial cells is stimulated by proinflammatory cytokines, and results in adhesion of endothelial cells to the vascular lining and accumulation of blood leukocytes at the sites of inflammation [38]. In our study, we found that compared to their normal counterparts, AMD plasma and AMD RPE cells expressed significantly elevated protein levels of E-Selectin (CD62E) by 77.1 % and 158.5 % respectively, indicating the involvement of E-Selectin in AMD-related inflammation. Next, we demonstrated notably higher P-Selectin (CD62P) protein levels in the AMD plasma and AMD RPE cybrid cells by 75 % and 198.9 % respectively, indicating the significant contribution of P-Selectin in ocular inflammation. Consistent with our results, a previous study reported elevated levels of P-Selectin are found in non-arteritic anterior ischemic optic neuropathy, indicating that increased P-Selectin is a pathological marker associated with this ophthalmologic disease [39]. P-Selectin was also elevated in diseases with an inflammatory component such as rheumatoid arthritis [40]. P-Selectin i.e., CD62P is encoded by the *SELP* gene and is localized to the α-granules of platelets and Weibel-Palade bodies of endothelial cells. In response to stimulation by thrombin or other agonists such as histamine or collagen, P-Selectin is translocated to the surface of activated platelets (thrombocytes) and activated endothelial cells, where it plays a critical role in the recruitment of leukocytes to the site of injury during inflammation. P-Selectin is composed of an N-terminal C-type lectin domain, an EGF (Epidermal-Growth-Factor)-like domain, a conserved complement regulatory domain consisting of nine short consensus repeats of cysteine residues, a transmembrane domain, and a cytoplasmic tail region [41–44]. A recent study that examined the expression of Selectins in the retina and choroid of AMD patients and compared the allele and genotype frequencies between AMD patients and controls, demonstrated that a single SNP (Single Nucleotide Polymorphism) located within an intron of *SELP* gene (rs3917751) is statistically associated with dry AMD in their study cohort. This was the only ancestral risk allele for dry AMD that was found in the P-Selectin gene [45]. Our current study revealed that treatment with HNG decreased the protein levels of both E-Selectin and P-Selectin by 64.62 % and 60.99 % in AMD RPE cybrid cells compared to normal, thereby indicating that Humanin G suppresses inflammation by downregulating E- and P-Selectins. This is an important finding since inhibition of E-Selectin using microRNAs suppresses endothelial cell inflammation [46].

Apart from Selectins, we also examined another cell adhesion molecule – ICAM-1 (Intercellular adhesion molecule-1). ICAM-1 i.e., CD54 (Cluster of Differentiation 54) encoded by the *ICAM-1* gene is a transmembrane glycoprotein that is typically expressed on endothelial cells and facilitates the binding of leukocytes to vascular endothelium [47]. In the current study, we found significantly increased protein levels of ICAM-1 in AMD plasma and AMD RPE cybrid cells by 83.6 % and 47.5 % respectively, and treatment with HNG reduced the ICAM-1 protein by 30.82 % in AMD RPE cybrid cells. Similar to our study, a previous study demonstrated that subfoveal choroidal neovascular membranes (CNVMs) surgically excised from AMD patients have higher protein levels of ICAM-1 compared with those in the normal eye [48]. Increased ICAM-1 protein levels were also found in the choroid and sclera of hypercholesterolemic New Zealand rabbits [49]. Our results are consistent with previous studies showing enhanced ICAM-1 protein levels in the aqueous humor samples of wet AMD patients and notable upregulation of retinal *ICAM-1* in a VEGF (Vascular Endothelial Growth Factor)-induced retinal leukostasis rat model. Inhibition of ICAM-1 bioactivity significantly reduced retinal leukostasis and vascular permeability, thereby indicating that ICAM-1 plays a key role in inflammation and VEGF-induced retinal leukostasis [50, 51]. Retinal leukostasis, a manifestation of retinal inflammation, is induced by pro-inflammatory cytokines and VEGF, and is characterized by an increase in the number of static leukocytes in the retina, enhanced adhesion of leukocytes and monocytes to retinal endothelial cells, reduced retinal blood flow, luminal narrowing of retinal capillaries, and decreased perfusion pressure [52–54]. Humanin G’s ability to reduce ICAM-1 protein is remarkable because targeted homozygous knockout of *ICAM-1* gene in a laser injury-induced choroidal neovascularization (CNV) mouse model notably inhibited CNV as evidenced by substantially diminished volume of CNV lesions and decreased fluorescein leakage in *ICAM-1* deficient mice compared to wild-type mice after laser photocoagulation injury [55]. In addition to the retina, ICAM-1 is involved in corneal inflammatory processes as well. ICAM-1-deficient mice show significant reduced neovascularization compared to controls, thereby indicating that ICAM-1 is a mediator of inflammation-associated and VEGF-induced corneal neovascularization [56]. Our results underscore the importance of cell adhesion molecules in AMD pathology and highlight the vital role of HNG in alleviating retinal inflammation in AMD.

Next, we tested the protein levels of cytokines and chemokines and investigated the effects of Humanin G treatment on the expression of the pro-inflammatory markers. TNF-α (Tumor Necrosis Factor alpha) is a key player in the pathogenesis of AMD as reduced TNF-α levels in the serum are associated with higher visual acuity score in AMD patients [57]. The transcription of *TNF-α* is genetically regulated and in the promoter region of the *TNF-α* gene, three SNPs were detected: *TNF-α-863* (rs1800630), *TNF-α-308* (rs1800629) and *TNF-α-238* (rs361525). Promoter polymorphisms at -238, -308, and -863 SNP positions could potentially regulate *TNF-α* production, and these polymorphisms may have implications for AMD pathogenesis due to an imbalance in inflammatory processes caused by dysregulation of TNF-α production [58–60]. *TNF-863CC/TNF-308GA* and *TNF-308GA/TNF-238GG SNP* genotypes combinations are associated with increased risk of AMD [61]. Only *-308 G/A TNF-*α gene polymorphism is associated with AMD. The *TNF-α* -1031 T/C polymorphism is significantly associated with wet AMD in the Taiwan Chinese population [62]. We report a 98.4 % increase in TNF-α protein levels in the plasma of AMD patients and a 111.3 % elevated TNF-α protein in the AMD RPE cybrid cells compared to their normal counterparts. This suggests the pivotal role of TNF-α in retinal inflammation in AMD. Consistent with our results, TNF-α protein levels were found to be significantly higher in the vitreous of AMD patients compared to that in normal individuals [63]. Significantly increased serum levels of TNF-α protein and associated retinal ischemia were found in Eales’ disease which is an idiopathic inflammatory retinal vasculopathy [64]. Moreover, TNF-α protein levels were markedly increased in the retina, RPE and choroid of Cxcr5 receptor-deficient mice [65]. Chronic exposure to TNF-α alters RPE morphology by interfering with RPE cell differentiation, development of transepithelial potential, and RPE tight junction formation, thereby resulting in RPE cells that resemble aged and diseased RPE cells found in AMD [66]. Higher levels of soluble TNF-receptor II protein have been found in the plasma of both early dry AMD and wet AMD patients, indicating systemic inflammation [67]. In our study, we discovered that addition of exogenous Humanin G to AMD RPE cybrid cells reduces the protein levels of TNF-α by 46.09 % compared to their untreated counterparts, thereby indicating that Humanin G could be used an effective inhibitor of TNF-α-induced ocular inflammation and might therefore alleviate AMD pathology. Although the effects of Humanin G on TNF-α in AMD have not been demonstrated before, along similar lines, it has been shown that administration of TNF-α inhibitors such as Adalimumab, a subcutaneous anti-TNF-α drug, in combination with anti-VEGF therapy improves visual acuity [68]. Intravitreal and intraperitoneal injections of Adalimumab prevent retinal degeneration and photoreceptor cell death by preventing TNF-α upregulation and reducing inflammation, oxidative stress, and apoptosis [69, 70].

Retinal inflammation, a major contributory factor in AMD pathogenesis, is characterized by chemokine-mediated infiltration of macrophages and microglia from the inner retina into the subretinal space. We found that the Monocyte Chemoattractant Protein-1 (MCP-1)/ CCL2 protein levels were significantly upregulated by 113.5 % in the plasma of AMD patients compared to normal plasma. This finding is important as MCP-1 is known to be secreted by RPE cells in response to oxidative stress damage and facilitates the migration and infiltration of macrophages and monocytes [71]. We next investigated the role of MIP-1α (Macrophage Inflammatory Protein-1α) i.e., CCL3 (C-C Motif Chemokine Ligand 3), a CC chemokine that is secreted by macrophages and binds to CCR1 and CCR5 chemokine receptors. MIP-1α mediates the recruitment of leukocytes and inflammatory cells to the site of inflammation in response to tissue injury. MIP-1α mediates wound healing and is involved in both cell-mediated immunity and systemic and mucosal humoral immune responses [72, 73]. We found a significant increase in MIP-1α protein levels by 185.2 % and 212.2 % in the AMD plasma as well as in the AMD RPE cybrid cells respectively, compared to that in the normal counterparts. Our results are consistent with previous studies that showed *MIP-1α* gene expression is notably upregulated and its function is nonredundant in retinal degeneration [74]. MIP-1α protein levels were remarkably elevated in aqueous humor samples obtained from the eyes of patients with exudative AMD and polypoidal choroidal vasculopathy, compared to that in control eyes. In addition, *MIP-1α* gene expression and protein levels were significantly upregulated due to post-ischemic pro-inflammatory response, in a mouse model of ischemic retinopathy [75, 76]. Humanin G-treated AMD RPE cybrid cells showed 61.98 % decreased MIP-1α protein levels compared with the untreated AMD RPE cybrid cells. Humanin G-induced suppression of MIP-1α might prevent AMD-associated ocular inflammation since previous studies have shown that inhibition of MIP-1α activity using neutralizing antibodies caused partial suppression of inflammation-induced retinal neovascularization, thereby indicating the critical contribution of MIP-1α in inflammation-induced retinal neovascularization [77]. MIP-1α plays a vital nonredundant role in chronic and acute inflammation-mediated retinal degeneration, and MIP-1α-deficient mice show reduced damage to the blood-retinal barrier compared with controls [65].

We demonstrated that AMD plasma and AMD RPE cybrid cells had 186.1 % and 137.3 % higher protein levels of IFN-γ (Interferon-gamma), which is secreted by the T Helper Type 1 (Th1) cells and its activation is a hallmark of adaptive and innate immune responses [78, 79], IFN-γ causes RPE cell death by increasing intracellular iron concentration, oxidative stress, lipid peroxidation, glutathione depletion, and activation of the JAK-STAT signaling pathway [80]. IFN-γ along with other cytokines induces the secretion of IL-6 from RPE cells [81]. In our study, addition of Humanin G reduced the IFN-γ protein by 62.86 % in AMD RPE cybrid cells compared to untreated AMD RPE cybrid cells. This highlights the potential of Humanin G to alleviate IFN-γ-mediated inflammatory responses in AMD, and it is important since IFN-γ plays a crucial role in the pathogenesis of AMD by: a) inducing the expression of *VEGF* in human RPE cells via the Phosphoinositide 3-kinase (PI3K)/mammalian target of rapamycin (mTOR) signaling pathway; b) enhancing mitochondria-generated ROS in human RPE cells; c) regulating the activation of the complement cascade; d) reducing complement inhibition; e) macrophage polarization; and f) diminishing the deposition of amyloid-β plaques in neuroinflammation [82–87]. Furthermore, Interferon gamma-inducible protein-10 (IP-10) i.e., C-X-C motif chemokine ligand 10 (CXCL10) is an α-chemokine that plays a key role in T cell adhesion to endothelial cells and as a chemoattractant to lymphocytes and monocytes [88]. We observed 193.5 % higher IP-10 protein levels in AMD plasma vs. normal plasma samples. This is consistent with recent studies which reported markedly elevated IP-10 levels in the postmortem eyes from dry AMD, geographic atrophy i.e., advanced AMD, and neovascular AMD patients [89]. Moreover, in our study, we found notably enhanced Interferon-alpha (IFN-α) protein levels by 133.3 % in AMD plasma samples compared to normal plasma. This finding suggests the involvement of IFN-α in AMD pathology since IFN-α causes retinopathy by inducing retinal vein occlusion, retinal ischemia, leukocyte infiltration, capillary non-perfusion, cotton wool spot formation, and accumulation of immune cells in the retinal vasculature [90, 91].

The IL-1β cytokine binds to the IL-1RI receptor and is an established biomarker for retinal diseases. It mediates the degeneration of rods by impairment of retinal glutamate homeostasis and severe loss of cone segments causing subsequent loss of visual acuity in AMD [92, 93]. IL-1β protein was notably increased by 330.6 % in the plasma and by 224.5 % in the cell lysates of AMD samples vs. controls, showing similar degree of involvement of IL-1β in both the AMD patients as well as AMD RPE cybrid cells. Our results are consistent with a previous study that demonstrated significant increase in the IL-1β protein levels in the serum of AMD patients [94]. Previous studies reported that both the ~31 kDa inactive pro-form and the cleaved ~17 kDa active form of IL-1β protein were significantly elevated in the vitreous samples obtained from patients with neovascular AMD and polypoidal choroidal vasculopathy, compared to controls [95–98]. Furthermore, *IL-1β* gene was markedly upregulated in the retinal vessels of diabetic retinopathy rats, and IL-1β protein levels were also elevated in the serum, vitreous fluid, and aqueous humor samples of diabetic retinopathy patients. These studies demonstrated a correlation between IL-1β and cell death in diabetic retinopathy [99–106]. Higher IL-1β protein levels are also associated with photoreceptor cell death and diminished visual fields in retinitis pigmentosa [107, 108]. Remarkably increased gene and protein levels of IL-1β were found in the blood and aqueous humor of glaucoma patients, establishing IL-1β as a risk factor in glaucoma pathogenesis [109]. In the retina and vitreous of patients with retinal detachments, higher IL-1β levels were reported [110, 111]. Macular edema also led to an increase in the IL-1β protein levels in the aqueous humor and vitreous samples [112, 113]. Elevated IL-1β levels were demonstrated in the aqueous humor and supernatants of posterior eye cups in an *in vivo* experimental autoimmune uveoretinitis (EAU) model, and administration of anti-IL-1β antibody remarkably decreased the EAU scores [114, 115]. Intravitreal injections of amyloid-beta, a component of the drusen in dry AMD, caused a remarkable increase in IL-1β protein in the vitreous of the treated rats compared with controls [116]. Moreover, in the *in vivo*, *ex vivo*, and *in vitro* models of retinopathy of prematurity, IL-1β was notably increased in the RPE/ choroid and caused choroidal involution, loss of RPE and photoreceptors, retinal degeneration, and visual deterioration. In addition, we found that exogenous Humanin G caused significant decrease in the IL-1β protein levels by 68.31 % in AMD RPE cybrid cells vs. untreated controls. Our results are novel as no previous study has demonstrated the Humanin G-mediated suppression of IL-1β protein in transmitochondrial AMD RPE cybrid cells. This finding is significant because inhibition of IL-1β using IL-1R receptor antagonist and recombinant IL-1Ra alleviates the damaging effects of IL-1β, markedly decreases subretinal neovascularization after laser injury, and substantially reduces photoreceptor cell apoptosis in AMD [117–120]. In addition, the expression of IL-1β gene and protein is upregulated several fold in rodent retinas following photo-oxidative damage and inhibition of IL-1β using siRNAs or neutralizing antibodies suppressed chemokine-induced inflammation and retinal degeneration in AMD [121, 122].

IL-13 is a 13 kDa cytokine secreted by T helper type 2 (Th2) cells, CD4 cells, natural killer T cell, basophils, and eosinophils among other cell types, and regulates allergic inflammation and IgE synthesis. In the current study, plasma from AMD patients had 336.5 % higher IL-13 protein levels whereas AMD RPE cybrid cells showed 177.6 % increase in IL-13 protein. Along similar lines, IL-13 protein levels were remarkably higher in the aqueous humor of AMD patients vs. controls, and IL-13 inhibited RPE cell proliferation [123]. Moreover, treatment of AMD RPE cybrid cells with Humanin G reduced IL-13 protein levels by 57.63 % compared to their untreated counterparts. This suggests Humanin G’s potential in suppressing IL-13-induced ocular inflammatory responses and is significant since inhibition of IL-13 reduces intraocular inflammation in acute-inflammation-induced retinal pathology [124].

IL-17 is a key cytokine produced by the T helper 17 (Th17) cells, which are derived from CD4+ cells. IL-17 is also secreted by CD8+ T cells, NK cells, and other immune cells and is involved in the neovascularization and pathogenesis of ocular diseases such as AMD, diabetic retinopathy, retinal vein occlusion, and retinopathy of prematurity. IL-17 causes ocular neovascularization by cytoskeleton remodeling, regulation of VEGF, and activation of complement components [125]. IL-17 mediates VEGF-induced angiogenesis by promoting the mitogenic activity of VEGF and VEGF-induced growth of vascular endothelial cells and triggers the secretion of IL-1β from RPE cells via activation of NLRP3 inflammasome [126, 127]. Similar to previous studies that have demonstrated notably elevated IL-17 protein levels in the serum of AMD patients and in RPE cells which constitutively expressed IL-17 receptors i.e., IL17 RA, IL-17RC, and ACT1 [128], we found that IL-17 protein levels were increased by 145.7 % in AMD plasma and were 101.4 % higher in AMD RPE cybrid cells compared to controls. Our findings corroborate the important role of IL-17 in AMD-associated inflammation and pathology since the expression of *IL-17A* and its receptor *IL-17RC* is markedly upregulated in the macular lesions of advanced-stage AMD donors compared to normal tissue [129]. IL-17A activates Caspase-3 and Caspase-9 pro-apoptotic proteins, thereby causing cytotoxicity in RPE cells. The expression of IL-17 and its receptor IL-17RC is upregulated in AMD eyes compared to controls [130]. IL-17A mediates blood-retinal barrier damage by activating the JAK1 signaling cascade, thereby leading to the disruption of tight junctions in RPE cells and endothelial cells [131]. IL-17 promotes the proliferation, migration, and tube formation in human choroidal endothelial cells by activating CCL2 and CXCL8 in RPE cells, thereby contributing to choroidal endothelial cell angiogenesis [132]. Next, we investigated the effects of Humanin G and discovered that treatment with Humanin G reduced IL-17A protein levels by 48.31 % in AMD RPE cybrid cells, thereby highlighting the ability of Humanin G to suppress IL-17-mediated retinal inflammation. This finding is important since it has been shown that knockdown of IL-17RC using siRNA reduces IL-17-mediated pathology in RPE cells [124].

IL-4 is a cytokine that mediates the differentiation and activation of T helper 2 cells and is a key regulator of humoral immune responses [133]. Polymorphisms of IL-4 -590 T/T or T/C genotypes is a potential genetic marker for the development of AMD pathology, and IL-4 promotes pathological angiogenesis and choroidal neovascularization, thereby leading to retinal degeneration [134, 135]. The 118.5 % upregulation of IL-4 protein in AMD plasma vs. normal plasma highlights its vital role in AMD-related inflammation.

IL-10 regulates macrophage functioning in the eye and promotes choroidal neovascularization in AMD. IL-10 deficiency alleviates choroidal neovascularization-induced damage and favors retinal health. [136] IL-10 protein was higher by 189.7 % in AMD plasma vs. normal plasma, consistent with previous studies and underlining its involvement in ocular inflammatory response in AMD.

IL-12p70 is primarily secreted by macrophages and dendritic cells and is a heterodimer comprised of p35 and p40 subunits. IL-12p70 which mediates the expression of IFN-γ and contributes to innate and adaptive immune responses, was found to have 107.7 % elevated protein levels in AMD plasma compared to normal plasma in our study. This underlines the crucial role of IL-12p70 in AMD pathology as it has been previously confirmed that IL-12p70 is upregulated in response to inflammation and oxidative stress in ARPE-19 cells [137].

To our knowledge, this is the first report that confirms the protective role of Humanin G against inflammation in AMD RPE transmitochondrial cybrid cells and it is significant because reducing ocular inflammation could alleviate its damaging effects observed in the RPE cells that eventually lead to retinal degeneration in AMD pathogenesis.

**Figure 6:**
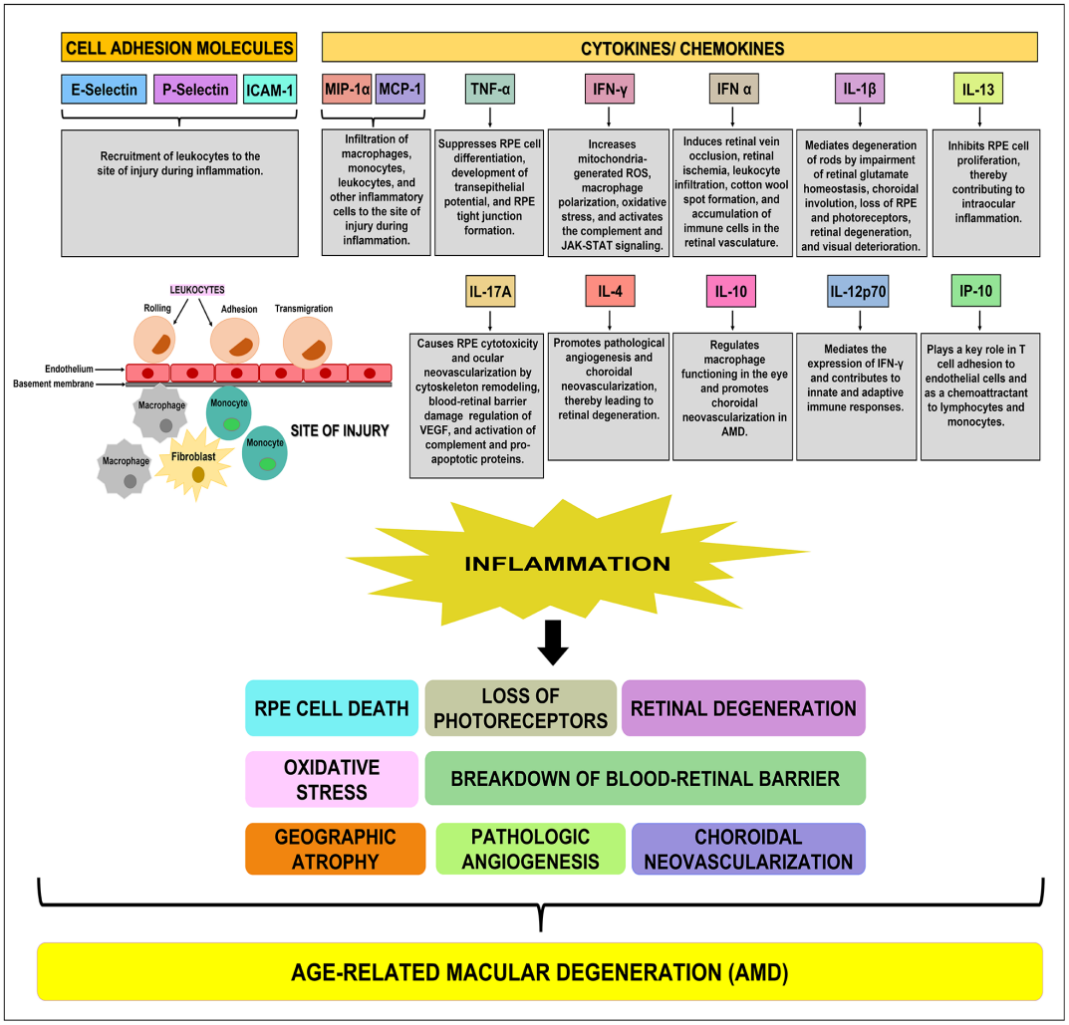
**Schematic showing the function(s) of cell adhesion molecules, cytokines, and chemokines.** E-Selectin, P-Selectin, and ICAM-1 are cell adhesion molecules that are involved in the recruitment of leukocytes to the site of injury during inflammation. MIP-1α, MCP-1, TNF-α, IFN-γ, IFN-α, IL-1β, IL-13, IL-17A, IL-4, IL-10, IL-12p70, and IP-10 are pro-inflammatory cytokines/ chemokines that promote RPE cell death, loss of photoreceptors, oxidative stress, retinal degeneration, breakdown of blood-retinal barrier, geographic atrophy, pathologic angiogenesis, and choroidal neovascularization, subsequently leading to the development of AMD.

**Figure 7:**
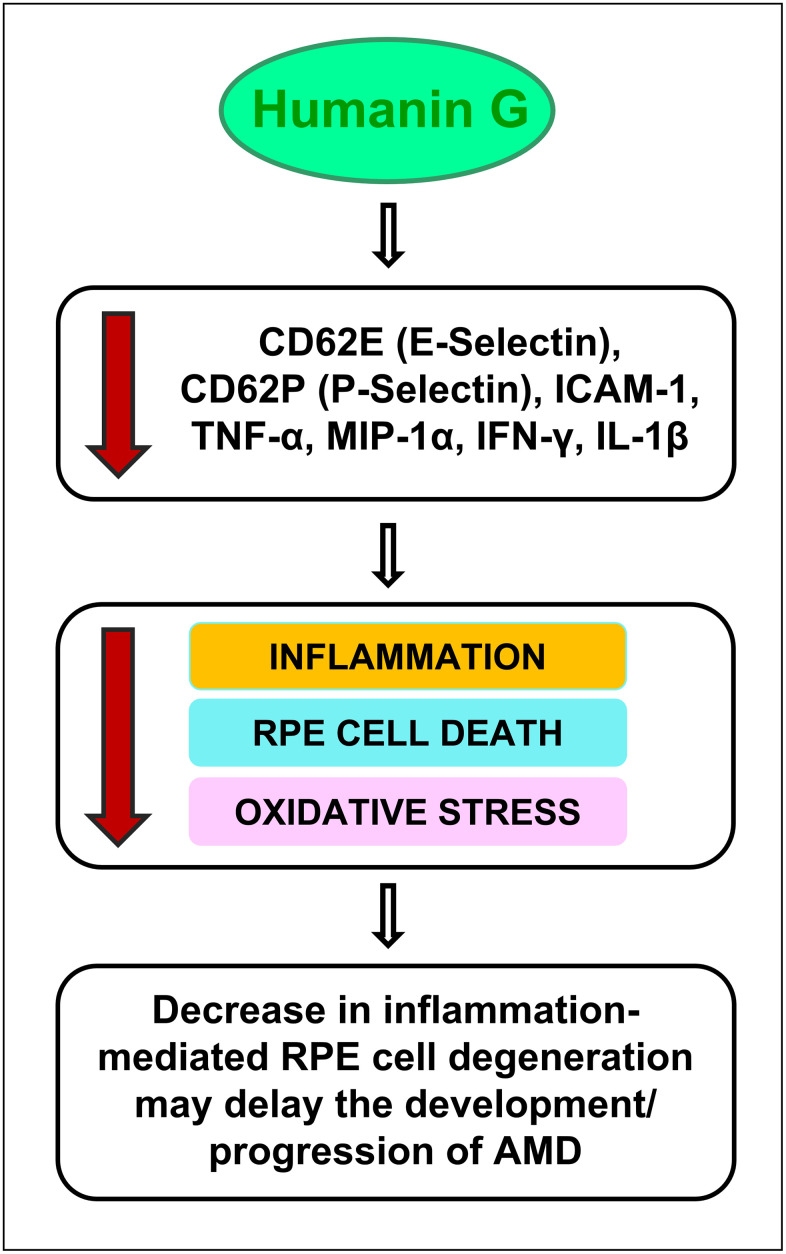
**Schematic showing the potential action of Humanin G.** Treatment with Humanin G reduces the levels of inflammation-associated proteins namely CD62E (E-Selectin), CD62P (P-Selectin), ICAM-1, TNF-α, MIP-1α, IFN-γ, and IL-1β. This is turn might decrease retinal inflammation, reduce RPE cell death and oxidative stress, thereby preventing retinal degeneration. This may delay the development/ progression of AMD.

In conclusion, our study reports novel findings that: A) demonstrate a decline in endogenous Humanin protein levels in plasma from AMD patients compared to normal subjects; B) underscore the role of cell adhesion molecules, cytokines, and chemokines in AMD-associated inflammation (Figure 6) and subsequent cellular damage in AMD RPE cybrid cells that has been observed in our previous studies; and C) establish the positive effects of Humanin G in reducing the expression of inflammatory markers in AMD RPE transmitochondrial cybrid cells and therefore demonstrate the potential of Humanin G in reducing AMD-associated inflammation *in vitro* (Figure 7)*.* Since we found elevated inflammatory proteins in the AMD plasma, which suggests that inflammation might be more systemic and not just confined to the retina, we speculate that a baseline systemic inflammation may make the retinal cells more susceptible to damage, thereby leading to AMD pathology.

It is important to note that in cells that are stressed/ damaged, Humanin G acts to suppress the inflammatory proteins that may contribute to the development of AMD. It is noteworthy that in the cells that are healthy and have a normal homeostasis, Humanin G does not exert any negative impact. This is significant because to be used successfully to treat diseases, you want the drug to be targeting the diseased cells but have no negative impact on the healthy cells.

Further studies are required to gain an in-depth understanding of the mechanisms underlying Humanin G-mediated suppression of inflammation in AMD and to establish Humanin G’s therapeutic potential as an inhibitor of AMD-associated inflammation. Furthermore, in addition to administration of Humanin G, knockdown or knock-out of the studied inflammatory markers using siRNA or CRISPR editing, may present a new line of treatment for AMD.
